# Bariatric Surgery in Asthma: A Narrative Review

**DOI:** 10.3390/medicina60050806

**Published:** 2024-05-14

**Authors:** Maciej Mawlichanów, Paulina Tatara, Andrzej Kwiatkowski, Anna Różańska-Walędziak, Maciej Walędziak

**Affiliations:** 1 Clinic of General, Oncological, Metabolic Surgery and Thoracic Surgery, Military Institute of Medicine in Warsaw, 04-141 Warszawa, Poland; 2 Dermatology Clinic, Military Institute of Medicine in Warsaw, 04-141 Warszawa, Poland; 3 Department of Human Physiology and Pathophysiology, Faculty of Medicine, Collegium Medicum, Cardinal Stefan Wyszynski University in Warsaw, 01-938 Warsaw, Poland

**Keywords:** asthma, obesity, asthma after surgery, surgery in asthma

## Abstract

Nearly 60% of asthmatics in the USA suffer from obesity. Asthma is a comorbid condition alongside obesity, commonly accompanied by conditions such as hypertension and type 2 diabetes. The positive effect of bariatric surgery on patients suffering from hypertension and type 2 diabetes, which leads to either a reduction in the dose of medication taken for the aforementioned diseases or the withdrawal of the disease, is quite well proven in the literature. Currently, the impact of bariatric operations on the control and course of bronchial asthma and pharmacological treatment has not been fully recognized and described, requiring further research; therefore, the following review of the literature was conducted.

## 1. Introduction

Bronchial asthma is a heterogeneous disease, usually characterized by chronic inflammation of the respiratory tract. Among the symptoms, the most common are wheezing, a feeling of breathlessness, chest tightness, and a cough of variable frequency and severity, as well as the obstruction of variable severity of expiratory airflow through the airways [1]. Over the years, the disease also leads to the remodeling of the bronchial walls. It is estimated that about 300 million people worldwide suffer from bronchial asthma [2], and it is a significant cause of chronic obstructive pulmonary disease, which is the fourth leading cause of death worldwide. It is currently the most common chronic disease in people up to the age of 40. Bronchial asthma is divided into four groups based on etiology: early-onset allergic asthma, moderate to severe early-onset allergic asthma with remodeling, late-onset eosinophilic non-allergic asthma, and late-onset non-allergic asthma [3]. One of the unique subtypes of non-allergic asthma is the phenotype associated with obesity [4], which typically has a worse course and poorer response to treatment. The immunological activity of adipose tissue in chronic inflammation processes is of significant importance. The diagnosis of bronchial asthma is made based on symptoms and the results of spirometric testing, where a significant improvement in forced expiratory volume in 1 s (FEV1) and/or forced vital capacity (FVC) according to Global Initiative for Asthma (GINA) by more than 12% and 200 mL [5] is seen during a bronchodilator test. Obesity is diagnosed when the Body Mass Index (BMI) is 30 or higher [6]. Bronchial asthma is most commonly differentiated from chronic obstructive pulmonary disease, in which case, spirometry is the most important test.

The analyses conducted so far have not found clear indications for bariatric surgery in the case of people suffering from bronchial asthma. Additionally, the possibility of using artificial intelligence to expand and standardize materials for analysis has not been considered so far. The authors hope that the following work will contribute toward strengthening the position of teams treating people with the conditions covered by this review.

## 2. Search Strategy

A literature review with an in-depth analysis of descriptions of observations to date was performed by two independent persons. The authors used available sources and articles from PubMed. Key search terms included “asthma after bariatric surgery”, “asthma in bariatric patient”, and “bariatric asthma”. The articles for this study spanned the years 2013–2023 and were selected carefully for information on the variability of the course of bronchial asthma in people after bariatric surgery. Papers describing the topic of asthma treatment and course in obese people in very general terms and those without bariatric surgery and case reports were rejected. The papers were divided into sections on dose reduction of anti-asthma medication, description of asthma disease according to scoring scales, biochemical factors studied, and need for emergency healthcare.

## 3. Reduction in Anti-Asthmatic Drug Doses after Bariatric Surgery

The treatment of bronchial asthma is implemented on the basis of its degree of control according to special scoring tables. The usual basis is inhaled steroid drugs in the lightest version and beta-antagonists, and oral corticosteroids in the most severe forms. In recent years, leukotriene antagonists and antibodies have gained popularity and recognition. In one retrospective cohort study, the behaviors of 320 patients before and after bariatric surgery were analyzed. It was proven that the number of medications taken by the patient significantly decreased after surgery, and symptoms were reduced. Analyses showed that the average (SD) number of prescriptions filled preoperatively was 7.0 (6.9) annually, dropping to 3.8 (6.1) annually in the first postoperative year [7]. Another study involving 751 patients confirmed the hypothesis that bariatric surgery significantly reduces the use of anti-asthmatic drugs as early as 30 days post-surgery. Over time, comparing 30 days, 180 days, and 365 days, the reduction in medications was declared by 27%, 37%, 44%, and 46% in the 3-year period, respectively [8]. A review and meta-analysis published in 2022 by Luyu Xie, after analyzing 13 other studies, proved that 54% of post-bariatric patients stop taking medications post-surgery [9] and there is a decrease in people taking medications from 49.8% to 29.6% [10].

## 4. Asthma Control and Quality of Life Based on Tests in Conjunction with Bariatric Surgery

Metabolic syndrome is a group of traits conditioned by test results such as waist circumference, hypertension, impaired fasting glucose or type 2 diabetes, and triglyceride levels [9]. Erick Forno et al. published the results of their observations that metabolic syndrome negatively affects asthma control according to the Asthma Control Test (ACT) questionnaire. Additionally, it negatively affects the effect of bariatric surgery. The presence of metabolic syndrome in patients undergoing surgery increased the loss of its control post-surgery [11]. A study also compared surgical weight loss versus non-operative methods on the effect on asthma control in addition to indicating a higher percentage of weight loss of 22–36% in conjunction with non-surgical techniques (4–14%) [12]. To provide a reliable assessment of asthma control, tests such as the Asthma Control Test, Asthma Control Questionnaire (ACQ), and mini Asthma Quality of Life Questionnaire (mini-AQLQ) are used. These are simple and easy tests that, after being filled out at least once in the presence of a doctor, the patient can perform independently and discuss the results with their attending physician. These tools provide the doctor with a reliable picture of the patient’s well-being without delving into spirometry parameters, which indirectly affect the patient’s well-being. Several years of observations comparing patients after bariatric surgeries with a group of patients who were not operated on indicate that in every time interval from surgery to 5 years, the results of the above tests consistently improved, indicating better quality of life and asthma control in individuals who opted for surgery [13]. However, in patients who participated in an 8-year study cited by Jan Witte, in addition to the improvement in the ACQ score from 0.4 to 0.7 after 12 months, a slight increase in weight between the 12th month and the 96th month (BMI 30.2 kg/m^2^ vs. 32.3 kg/m^2^) was shown, which did not significantly affect the ACQ results [14]. Patients after bariatric surgeries are almost half as likely to appear in the hospital emergency department due to exacerbations of the underlying disease [15]. According to the article “Long-term improvements in pulmonary function 5 years after bariatric surgery”, 48% of respondents have no asthma symptoms 5 years after surgery [16].

## 5. Spirometric and Biochemical Indicators Studied in Obese Asthmatics

In a routine spirometric examination, factors such as FEV1 (forced expiratory volume in the first second), FVC (forced vital capacity), FEV1/FVC, i.e., the Tiffeneau test, Maximal Expiratory Flow (MEF) or Forced Expiratory Flow (FEF) 25, 50, 75 indices, and Peak Expiratory Flow (PEF) are assessed. The most important indicators affecting the diagnosis of bronchial asthma are FEV1 and FVC, which are the subject of research. In a 5-year follow-up, FEV1 increased by 4.1% in women and 6.7% in men after bariatric surgery. FVC increased by 5.8% in women and 7.6% in men. Gender and weight loss were independently associated with the improvement in FEV1 and FVC [13]. Since both FEV1 and FVC increased, the FEV1/FVC ratio did not change compared to the control group. In a certain group of patients, the acute-phase protein C-Reactive Protein (CRP) index was examined before bariatric surgery and 6 and 12 months after. The decrease from 8.6 to 1.7 mg/L, along with the above, led to an improvement in airway reactivity [17,18]. It seems that the evaluation of biochemical inflammatory markers, or, rather, their fluctuation in relation to body weight, could be an important line of research. Inflammatory parameters such as CRP and Tumor Necrosis Factor (TNF) vary depending on body weight. Weight loss is associated with significant changes in inflammation profiles, both for anti-inflammatory factors such as interleukin-4 and pro-inflammatory ones such as TNF-a [19] or the expression of interleukin-6, tumor necrosis factor superfamily member 14 (TNFSF14) (also known as LIGHT), Lymphotoxin beta receptor (LTBR), Matrix Metalloproteinases-9 (MMP-9), and C-Chemokine Receptor type 2 (CCR-2) [20].

## 6. Asthma and Obesity

One of the phenotypes of asthma is associated with obesity [4]. So far, no single factor has been proven or indicated to explain why so many obese individuals suffer from asthma and what factor determines it. However, it is observed that weight reduction clearly contributes to better control or withdrawal of asthma. Reduced exacerbations, according to Peters U et al., may be related to effects on lung mechanics and airway reactivity. The poorer results of the treatment of viral or bacterial infections in obese individuals and their poorer response to vaccination suggests that any loss of weight may help avoid the development of asthma [19]. Obese patients are more often hospitalized for bronchial asthma and use more anti-asthmatic drugs compared to asthmatics without obesity. It is noted that the treatment of asthma in obese patients should be comprehensive, as the body’s response to drugs is weaker compared to a lean asthmatic patient. Weight reduction is a remedy, even if achieved invasively (surgically) [21].

## 7. Surgery as a Potential Treatment for Asthma

After analyzing many studies and articles, the question arises as to whether surgical bariatric treatment could be a way to treat bronchial asthma. The results of many studies emphasize the uncertain, yet significant, role of bariatric surgeries in this disease. However, the studies have in common a small number of patients, incomplete follow-up, different time frames, a lack of standardization, single-center studies, and other limitations that do not allow for a definitive conclusion. Usually, each of the analyzed studies possesses one of the above features. A mechanism is being sought to provide a clear answer as to whether surgical treatment can be effective against asthma [22]. Additionally, the elasticity of the airways [23,24,25] is considered. A significant role may also be played by mechanical factors associated with weight loss, as it has been shown that in obese patients, breathing with small air volumes causes a reduction in Functional Residual Capacity (FRC) and Expiratory Reserve Volume (ERV), which leads to airway collapse [22,26].

## 8. Perspectives for the Future

Considering that obesity is a civilizational disease that is becoming increasingly prevalent, and we are witnessing an increase in the number of obese people from an early age, the problem of bronchial asthma will be an increasingly common issue in the offices of family doctors, internists, surgeons, and emergency medicine physicians. Given the increasing number of bariatric patients, the ability to adjust treatment and predict its consequences seems to be a decidedly interdisciplinary skill. Perhaps at each stage of prevention, qualification, and bariatric treatment, knowledge regarding asthma treatment, but also the proposal of bariatric treatment for patients with asthma as one of the treatment alternatives, will be required. Bariatric surgeries may become a preventive measure as well as a remedy for diseases previously considered strictly internal, especially in the case of unsuccessful conservative treatment. Perhaps bariatric surgery will find its official indication as a method of treating bronchial asthma in the future. However, this requires further in-depth research and analysis, which is why the authors conducted this review. It seems that artificial intelligence could be revolutionary, gathering data for the doctor on each patient from an integrated network of hospitals and their contacts with healthcare, about a prescription filled for medications, or automatically including the patient in the observational study database after surgery. 

## 9. Surgery Is an Invasive Method

It should be remembered that bariatric surgery is an invasive method that has its limitations and risks. Not every patient can be operated on and restrictions often depend on the country and the guidelines of national societies. Bariatric surgery is an invasive field and even though it is called minimally invasive, complications may occur as frequently as during other standard surgical procedures. The undoubted advantage is that we can often eliminate the problem definitively, but the disadvantage is the operational risk.

## 10. Conclusions

Based on the analyzed material, it can be concluded that the surgical treatment of individuals with obesity may improve the treatment outcomes of bronchial asthma. It was indicated that the number of people admitted to the hospital emergency department due to the exacerbation of asthma decreases, FEV1 and FVC parameters increase, reduced doses of anti-asthmatic drugs allow for good asthma control, and there is an improvement in health status reflected in the above scales. As of the first quarter of 2024, too few studies have been conducted and described to unequivocally support this paper, hence the need for the involvement of many centers to perform their analyses. It seems sensible to understand that bronchial asthma is an interdisciplinary disease, and positive effects could be achieved by tightening cooperation between surgery departments performing bariatric surgeries and pulmonary clinics, allergology clinics, and family doctor offices in order to achieve better treatment outcomes for patients. If it is confirmed beyond doubt that surgical treatment improves asthma management, this indicates that every patient from the allergy clinic should be referred for consultation to the bariatric clinic, and vice versa.

## Data Availability

Not applicable.
